# Correlation between small-cell lung cancer serum protein/peptides determined by matrix-assisted laser desorption/ionization time-of-flight mass spectrometry and chemotherapy efficacy

**DOI:** 10.1186/s12014-024-09483-8

**Published:** 2024-05-19

**Authors:** Zhihua Li, Junnan Chen, Bin Xu, Wei Zhao, Haoran Zha, Yalin Han, Wennan Shen, Yuemei Dong, Nan Zhao, Manze Zhang, Kun He, Zhaoxia Li, Xiaoqing Liu

**Affiliations:** 1 Department of Oncology, PLA Rocket Force Characteristic Medical Center, Beijing, 100088 China; 2 Department of Oncology, The Fifth Medical Center of PLA General Hospital, Beijing, 100071 China; 3 National Center of Biomedical Analysis, Beijing, 100850 China

**Keywords:** Small-cell lung cancer, Matrix assisted laser desorption ionization-time of flight-mass spectrometry, Proteomics, Chemotherapy, Prediction of efficacy

## Abstract

**Background:**

Currently, no effective measures are available to predict the curative efficacy of small-cell lung cancer (SCLC) chemotherapy. We expect to develop a method for effectively predicting the SCLC chemotherapy efficacy and prognosis in clinical practice in order to offer more pertinent therapeutic protocols for individual patients.

**Methods:**

We adopted matrix-assisted laser desorption/ionization time-of-flight mass spectrometry (MALDI-TOF-MS) and ClinPro Tools system to detect serum samples from 154 SCLC patients with different curative efficacy of standard chemotherapy and analyze the different peptides/proteins of SCLC patients to discover predictive tumor markers related to chemotherapy efficacy. Ten peptide/protein peaks were significantly different in the two groups.

**Results:**

A genetic algorithm model consisting of four peptides/proteins was developed from the training group to separate patients with different chemotherapy efficacies. Among them, three peptides/proteins (m/z 3323.35, 6649.03 and 6451.08) showed high expression in the disease progression group, whereas the peptide/protein at m/z 4283.18 was highly expressed in the disease response group. The classifier exhibited an accuracy of 91.4% (53/58) in the validation group. The survival analysis showed that the median progression-free survival (PFS) of 30 SCLC patients in disease response group was 9.0 months; in 28 cases in disease progression group, the median PFS was 3.0 months, a statistically significant difference (χ^2^ = 46.98, *P* < 0.001). The median overall survival (OS) of the two groups was 13.0 months and 7.0 months, a statistically significant difference (χ^2^ = 40.64, *P* < 0.001).

**Conclusions:**

These peptides/proteins may be used as potential biological markers for prediction of the curative efficacy and prognosis for SCLC patients treated with standard regimen chemotherapy.

## Contribution to the field

we sought to identify reliable biomarkers for prediction of the curative efficacy and prognosis for small-cell lung cancer (SCLC) patients treated with standard regimen chemotherapy. For this purpose, we analysed serum samples from SCLC patients using the ClinPro system combined with matrix-assisted laser desorption/ionization time-of-flightmass spectrometry (MALDI-TOF-MS). Using the ClinPro software and a genetic algorithm analysis, a panel of serum markers that efficiently predicted the the curative efficacy of SCLC chemotherapy.

## Background

Lung carcinoma, also known as primary bronchogenic carcinoma, is a highly malignant tumor with poor prognosis [1]. As one of the most common malignant tumors, its incidence continues to rise in recent years. Small-cell lung cancer (SCLC) accounts for approximately 15-20% of lung cancers diagnosed each year and 25% of deaths from lung cancer [2, 3]. Most SCLC patients are unable to be cured through surgery because of invasive growth, rapid proliferation, early metastasis and recurrence. Chemotherapy is a basic method in the treatment of SCLC and occupies an important position in therapeutic options. Currently, the standard first-line chemotherapy recommended by guidelines is a regimen of etoposide and platinum-based medications. The response rate (RR) of this regimen is relatively high, up to 70-90% for localized-stage SCLC and up to 50-60% for extensive-stage SCLC [4]. However, approximately 20% of localized stage patients and 30% of extensive stage patients experience disease progression during or shortly after first-line chemotherapy [5–7]. The failure of first-line treatment has significant adverse effects on patient survival. The major reason for treatment failure is considered to be related to primary drug resistance. In contrast to the dramatic progress in the treatment of non-small-cell lung cancer (NSCLC), cytotoxic chemotherapy remains a basic treatment for SCLC. Therefore, enhancing the RR of chemotherapy is essential to improve the prognosis of SCLC. Nevertheless, there no methods or techniques to effectively monitor the efficacy of chemotherapy and predict prognosis. In addition, histopathological examination and disease stage classification cannot provide more information relevant to prognosis. Accordingly, there is an urgent requirement to develop a method for effectively predicting the SCLC chemotherapy efficacy and prognosis in clinical practice in order to offer more pertinent therapeutic protocols for individual patients. The proteomic technology developed in recent years has opened a new chapter for tumor markers. The mass-spectrometric technique, as the core of proteomic technology, has been rapidly developed in basic and clinical research. The mass-spectrometric technique for the analysis of analyze serum samples can detect plasma/serum proteins in sub-groups of patients with the same disease. Taguchi [8] and Wu [9] used the mass-spectrometric technique on plasma and serum samples from non-small-cell lung cancer (NSCLC) patients and found minor differences in the plasma/serum proteins between two sub-group patients who had significantly different efficacies of treatment with epidermal growth factor receptor tyrosine kinase inhibitors (EGFR-TKIs). Furthermore, they used the different peptides/proteins to establish a classification model to judge or predict the efficacy of EGFR-TKI treatment. These outcomes encouraged the investigators to explore the potential biological markers using proteomic technology for predicting the efficacy of SCLC chemotherapy.

The MALDI-TOF-MS technique adopted in this study incorporated the techniques of MALDI-MS and TOF, which are used to study small molecular substances with high sensitivity, high accuracy and high resolution [10, 11]. This technique has been widely used in many oncology studies [12–14] and is also actively used in basic and clinical research studies of lung carcinoma [15–17]. This study adopted the MALDI-TOF-MS technique to detect the differences in serum peptides/proteins between two groups of patients with contrasting SCLC first-line chemotherapy efficacy and then screened out the different peptides/proteins with ClinPro Tools software to design a classification model for predicting the SCLC chemotherapy efficacy. The model was verified by SCLC patients’ serum samples in the validation group through a blinded mode. We also conducted survival analyses for patients in the validation group who were classified by the model to verify the application potential of this model for the prediction of the prognosis in SCLC patients.

## Materials and methods

### Patients and sample

A total of 154 pre-treatment serum samples were obtained from the Department of Lung Cancer of the 307 Hospital of PLA between June 2012 and June 2014. The inclusion criteria were as follows: pathologically confirmed SCLC, an Eastern Cooperative Oncology Group (ECOG) performance status of 0 or 1, older than 18 years of age, and without severe underlying diseases (heart, liver and kidney). This study was performed according to protocols approved by the local ethical committee (the Ethics Committees of The Fifth Medical Center of PLA General Hospital), and all patients provided written informed consent to participate and permission to use their blood samples. For the tumor response assessment, we evaluated objective responses after 8 weeks of treatment based on computed tomography (CT) scans. The tumor response was determined according to the evaluation criteria in solid tumours (RECIST vision 1.1). The overall survival (OS) was defined as the time from the date of lung cancer diagnosis to the date of death. The progression-free survival (PFS) was defined as the time from the start of EGFR-TKI treatment to the date of disease progression or death from any cause. Smoking status was based on records from the patient’s initial visit to the clinic; smokers were defined as having smoked more than 100 year-cigarettes (the number of cigarettes in one day, multiplied by the Years of smoking).

After the collection of sera before treatment, the patients completed all relevant examinations and received the standard first-line treatment of a combinative chemotherapy regimen of platinum-based drugs and etoposide (VP-16). Adverse events after chemotherapy were all treated following regular clinical practice. Enrolled patients received the first-line chemotherapy for at least 2 cycles and the imaging examination was taken between the cycles to evaluate the therapeutic efficacy; first-line chemotherapy should not exceed 6 cycles. The cut-off date for follow-up was August 15, 2019.

According to RECIST (vision 1.1) standard, 50 samples from the patients were evaluated as complete response (CR) or partial response (PR) after first-line chemotherapy (referred to as Disease response group I), and 46 samples from the patients with progressive disease (PD) (referred as Disease progression group I below) were randomly selected to form the training group for the detection of differences in serum peptides/proteins between the two groups, and the generation of the classification mode, and the remaining patients composed of 30 patients with CR or PR (referred as Disease response group II below) and 28 patients with PD (referred as Disease progression group II below) formed the validation group to test the model.

Whole blood samples (5 ml) were collected before patients received the first-line therapy in a test tube, and blood was allowed to clot at room temperature for 1 h. After centrifugation at 3000 rpm for 10 min at 4 °C, serum was divided into aliquots and immediately stored at − 80 °C until later use.

### Peptidome isolation

We used weak cation exchange magnetic beads (MB-WCX, National Center of Biomedical Analysis, China) to fractionate serum samples following the standard protocol recommended by the manufacturer (Bruker Daltomik GmbH). Step 1, Binding. We mixed 20 μl binding solution (National Center of Biomedical Analysis, China), 5 μl MB-WCX beads that had been washed three times in 50 μl binding solution and 5 μl serum in a polymerase chain reaction tube, which was incubated for 10 min at room temperature. Step 2, Washing. We separated the unbound solution with a magnetic bead separation device, and beads were washed three times with 100 μl washing solution (National Center of Biomedical Analysis, China). Step 3, Elution. Bound proteins/peptides were eluted from the magnetic beads with 20 μl eluting solution (National Center of Biomedical Analysis, China) for MALDI-TOF-MS analysis.

### MALDI-TOF-MS analysis

For MALDI-TOF-MS analysis, 1 μl peptide eluate was mixed 1:1 (v/v) with a matrix solution that consisted of saturated α-cyano-4-hydroxy-cinnamic acid (α-HCCA, Bruker Daltonics, Germany) in 50% acetonitrile (ACN, Sigma–Aldrich, USA) and 0.1% trifluoroacetic acid (TFA, Sigma–Aldrich, USA) was spotted onto the sample anchor spots of an AnchorChip 600/384 target plate (Bruker Daltonics, Germany), which was allowed to air-dry at room temperature to let the matrix crystallize. ClinPro Peptide Calibration Standard I (Bruker Daltonics, Germany), a commercially available mixture of protein/peptides calibrators that consisted of angiotensin II (m/z 1,047.19), angiotensin I (m/z 1,297.49), substance P (m/z 1,348.64), bombesin (m/z 1,620.86), ACTH clip 1–17 (m/z 2,094.43), ACTH clip 18–39 (m/z 2,466.48), and somatostatin (m/z 3,149.57), was mixed 1:1 (v/v) with matrix solution, and 0.5 ml of the mixture was deposited on the calibrant anchor spots of an AnchorChip target plate for instrument calibration.

Mass spectrometry analyses were performed on an Ultraflex III MALDI-TOF-MS (Bruker Daltonics, Germany). The operating conditions were as follows: linear positive ion mode; repetition rate, 200 Hz; ion source voltages, 25 and 23.50 kV; lens voltage, 6.5 kV; and pulsed ion extraction time, 100 ns. For matrix suppression, we used a high gating factor with signal suppression of up to 300 m/z. For each spectrum, 3000 shots were manually acquired from six random positions over the surface of each spot (i.e., 500 shots per position). Data acquisition was carried out at 43% of the maximum laser energy. Each spectrum was externally calibrated. Peaks in the m/z range of 800–10,000 Da were recorded using FlexControl acquisition software v3.4 (Bruker Daltonics, Germany).

### Bioinformatics

#### Spectral processing

ClinPro Tools software v2.1 (Bruker Daltonics, Germany) was used to automatically process MALDI-TOFMS spectra data using data preparation settings according to the following standard workflow. Each raw spectrum was normalized to its total ion current. All spectra were recalibrated using the prominent, common m/z values. Next, baseline subtraction, smoothing, and peak detection were performed, and the peak areas for each spectrum were calculated. The signal-to-noise ratio was set at 5 for peak detection. Peak areas were calculated using zero level integration type. Spectra were also ‘‘top hat’’ baseline subtracted from the minimum baseline width set to 10%, and then were smoothed and processed in the 800–10,000 Da range.

#### Establishment of a training and classification model

Spectra from the training groups were used to build a classification model. Differential peptides peaks between the 50 patients from Disease response group Iand 46 patients from Disease progression group I were selected using peak areas that exhibited statistically significant differences. The built-in mathematical model’s Genetic algorithm (GA), Supervised Neural Network (SNN) and quick classifier algorithm (QC) were used to select each peptide peak and classification models were setup using ClinPro Tools 2.1 software to determine the optimal separation planes between samples from the two training groups. After each model was generated, a random cross-validation process was performed with the software, and the percent to omit and the number of interactions were set at 20 and 10, respectively.

To determine the accuracy of the class prediction model, the software offers cross validation and recognition capability. Cross validation is a measure of the reliability of a calculated model and can be used to predict how a model will behave in the future. This method is used to evaluate the performance of a classifier for a given data set under a given parameterization. Recognition capability describes the performance of an algorithm, i.e., the proper classification of a given data set.

#### Blind test of the classification model

The separated samples of the validation groups from 30 patients of Disease response group IIand 28 patients of Disease progression group II were used to show the accuracy of the classification model. Validation was performed in a blinded manner, as that MALDI-MS analysis was performed and classifications were labelled before clinical outcome data were made available to the investigators. For each sample from the validation groups, a corresponding spectrum was presented to the selected classification model. Then, the software returned a result that was compared with the actual efficacy.

### Statistical analysis

Comparisons of the clinical characteristics and the positive rate between different groups were made using the χ^2^ or Fisher’s exact test. Statistical analyses were performed using the SPSS software v19.0 (SPSS Inc., USA). A *p*-value less than 0.05 was considered to indicate a statistically significant difference. Comparisons of the area under the peptide peaks between different groups were made using the t-test with ClinPro Tools software (version 2.1). Kaplan-Meier (KM) estimate was used to simulate patients’ PFS and OS, as well as to draw the survival curves. Log-rank test was used to analyze the differences between curves.

## Results

### General characteristics of enrolled patients

A total of 154 SCLC patients were enrolled in this study. Eighty cases received curative efficacy as CR or PR after first-line standard chemotherapy, and the other 74 cases reached PD after treatment. According to the defined grouping principles, the enrolled patients were randomized into the following 2 groups: a training group and a validation group. The patients were balanced between the training and validation groups; the clinical and disease characteristics of the two groups are listed in Table 1.

**Table 1:** Clinical and disease characteristics of patients in the training and validation groups

	Training group (*n* = 96)			Validation group (*n* = 58)		
Characteristics	Disease response group I (*n* = 50)	Disease progression group I (*n* = 46)	*P* value	Disease response group II (*n* = 30)	Disease progression group II (*n* = 28)	*P* value
Age(years)			0.416			0.522
Mean Age ($(\bar x \pm s)$)	59.52 ± 10.88	60.01 ± 10.09		59.37 ± 11.15	60.50 ± 11.32	
Median (IQR)	(55.00, 68.00)	(55.75, 69.25)		(49, 69)	(51.25, 69.75)	
Gender, No. (%)			0.295			0.586
Male	32(64%)	34(74%)		15(50%)	16(57%)	
Female	18(36%)	12(26%)		15(50%)	12(43%)	
Smoking history, No. (%)			0.092			0.786
Smokers	30(60%)	35(76%)		15(50%)	15(54%)	
Non-Smokers	20(40%)	11(24%)		15(50%)	13(46%)	
Disease Stage, No. (%)			0.902			1.0
Limited	4(8%)	4(9%)		3(10%)	2(7%)	
Extensive	46(92%)	42(91%)		27(90%)	26(93%)	
ECOG PS, No. (%)			1.0			0.415
0–1	45(90%)	40(87%)		28(93%)	24(86%)	
2	5(10%)	6(13%)		2(7%)	4(14%)	
PFS(Months)	8.0	3.0	< 0.001	9.0	3.0	< 0.001
OS(Months)	13.0	7.0	< 0.001	13	7.0	< 0.001

ECOG PS: Eastern Cooperative Oncology Group Performance Status; PFS: progression free survival; OS: overall survival

### Differences in serum peaks of SCLC patients and healthy individuals in the training group

**Fig. 1:**
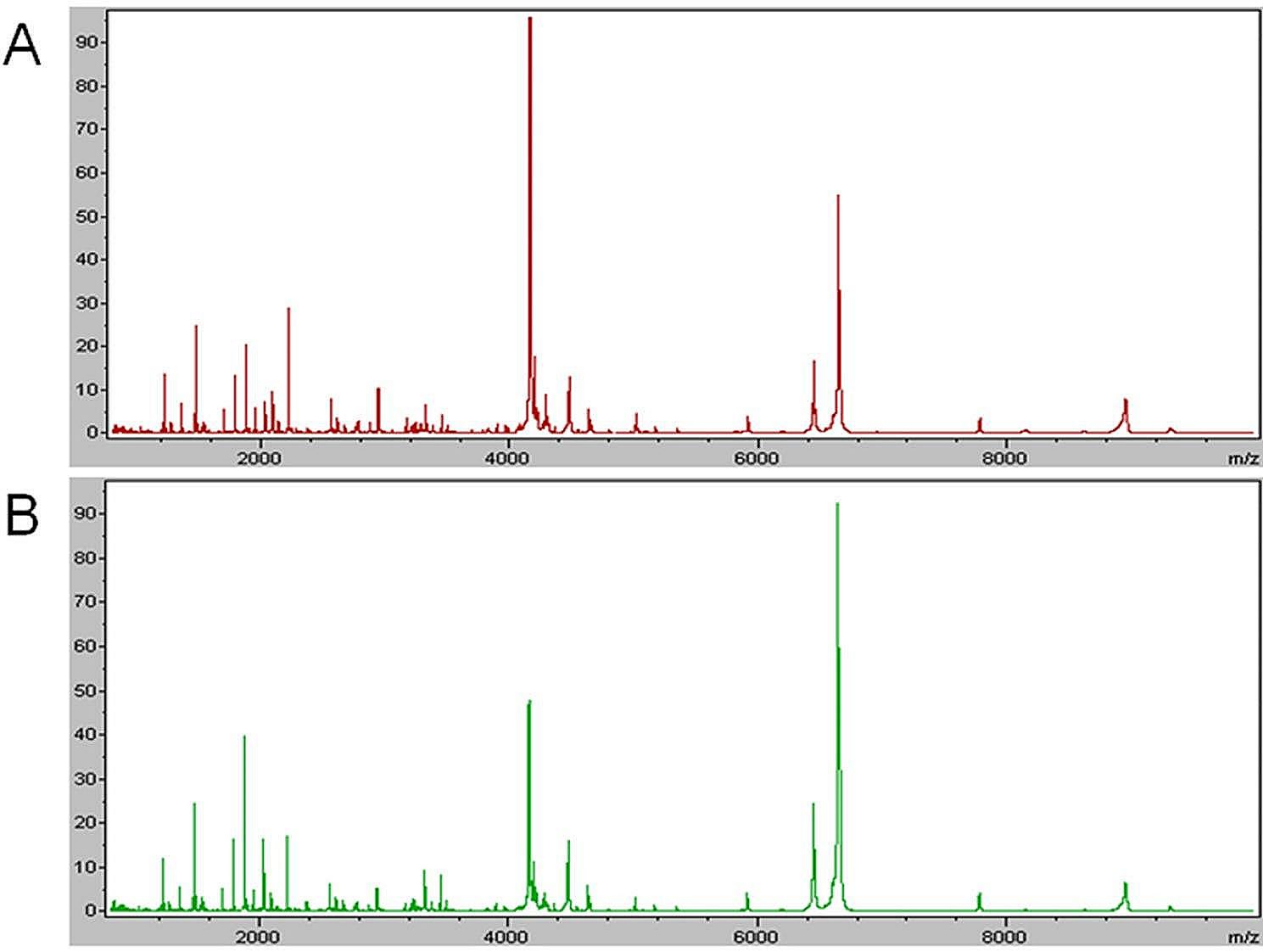
The average spectra of the training set displayed in ClinPro tools. (**A**) Average spectra for disease response group I in the training group. (**B**) Average spectra for disease progression group I in the training group

The training group included 50 SCLC patients with CR or PR responses (Disease response group I) and 46 patients with PD response after first-line chemotherapy (Disease progression group I), and the total average peptide spectra of the groups were analyzed using ClinPro Tools software (Fig. 1). Forty-four peptide peaks were identified in the spectra of the training group data set that was generated by MALDI-TOF-MS, and 10 peaks were significantly different (*p* < 0.0001, AUC ≥ 0.75) between the two groups (Table 2). In Disease progression group I, 3 signals exhibited a lower peak area and 7 signals exhibited a higher peak area than in Disease response group I.

**Table 2:** The 10 differential peaks in serum from Disease response group I and Disease progression group I in the training group

Mass-to-charge ratio	Disease response groupI (*N* = 50) (average peak area ± SD)	Disease progression group I (*N* = 46) (average peak area ± SD)	*P* Value	AUC	Expression level change in disease progression group I vs. Disease response group I
3323.35	22.23 ± 7.15	43.92 ± 13.49	<0.000001	0.96	↑
6649.03	224.26 ± 90.42	639.69 ± 264.91	<0.000001	0.99	↑
3224.24	9.45 ± 3.37	14.53 ± 4.9	0.00000387	0.84	↑
6795.74	3.56 ± 1.53	5.45 ± 1.71	0.00000515	0.82	↑
3158.66	28.8 ± 12.95	17.44 ± 5.81	0.00000761	0.8	↓
6780.03	4.42 ± 1.86	6.81 ± 2.34	0.00000761	0.81	↑
4283.18	83.22 ± 52.51	40.11 ± 26.88	0.0000399	0.76	↓
6451.08	78.17 ± 49.07	178.34 ± 124.48	0.0000586	0.82	↑
2082.49	54.74 ± 39.14	26.93 ± 18.99	0.000211	0.80	↓
6750.96	6.18 ± 2.83	9.79 ± 4.24	0.0000704	0.80	↑

### Establishment of a classification model

**Fig. 2:**
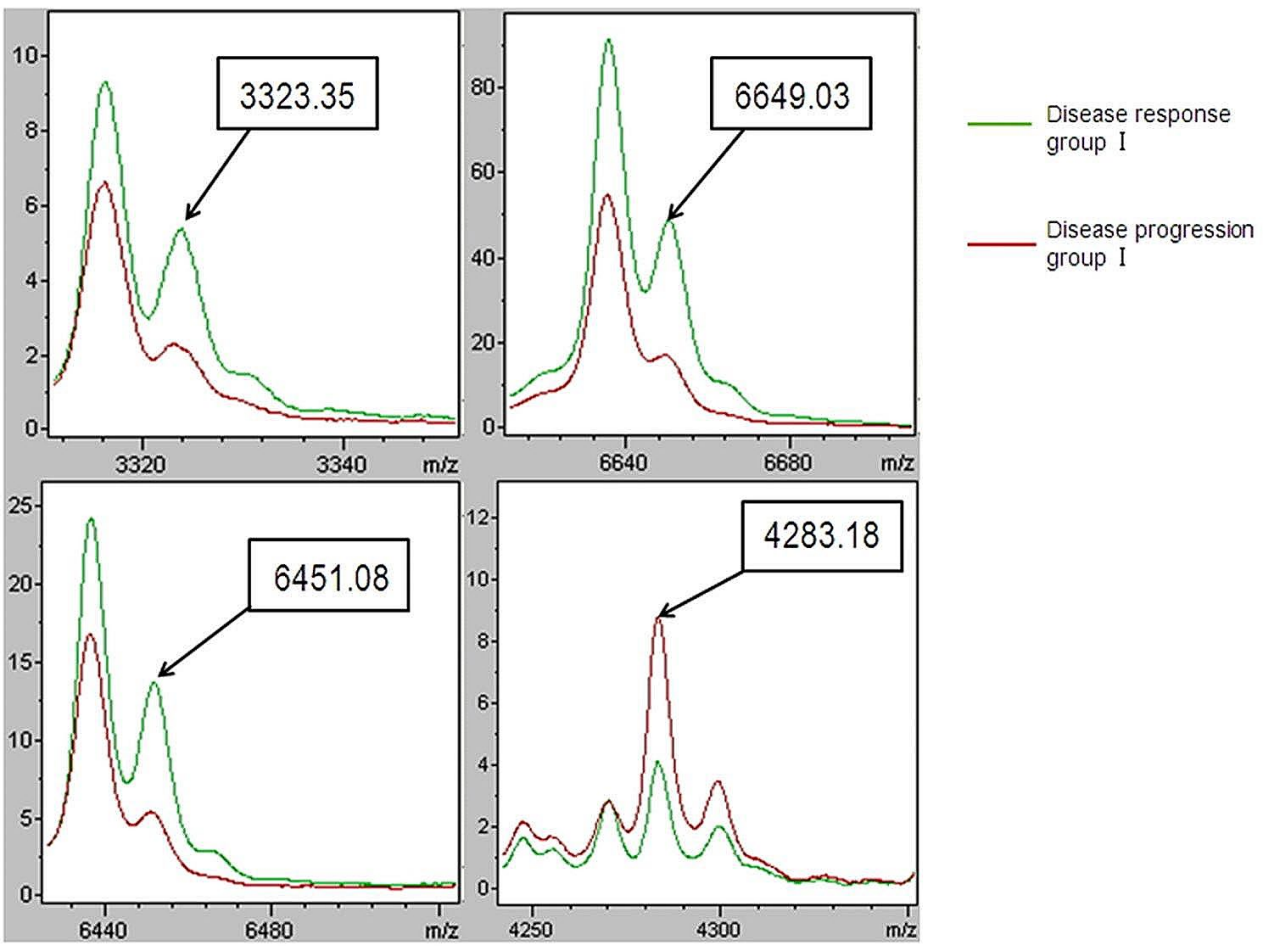
ClinPro Tools image of the average intensity, in arbitrary units, of four peptides that represent the classifier in disease response group I and disease progression group I. (Red curve: Disease response group I; green curve: Disease progression group I)

Three algorithms, GA (optimized by adjusting the number of neighbors for a k-nearest neighbor classification), SNN and QC, were applied for classification model construction using spectral data from the training group that was generated by MALDI-TOF-MS. By comparing the recognition capability and cross-validation of the models, we generated the optimal model-adopted GA algorithm. This model was composed of four peptide peaks at m/z 3323.35, 6649.03, 6451.08 and 4283.18 and exhibited the best efficiency in separating samples from Disease response group I and Disease progression group I, with a recognition capability of 99.00% and a cross-validation capability of 87.97% (Table 3; Fig. 2).

**Table 3:** Results of CPT statistical software for diagnostic model establishment

Model name	Cross validation rate	Recognition rate	Protein/peptides for model set up
GA	87.97%	99.00%	3323.35, 6649.03, 6451.08, 4283.18

### Blinded test of the classifier in the validation group

The classifier was then validated using an independent validation group of 30 patients from Disease response group II and 28 patients from Disease progression group II in a blinded test (Table 4). Among the 30 samples from Disease response group II, 27 (90.0%) were labelled as “Disease response group II” by the serum proteomics classifier, whereas among the 28 samples from Disease progression group II, 26 (92.9%) were labelled as “Disease progression group II”, achieving an overall accuracy of 91.4% (53/58).

**Table 4:** Validation results of prediction model for curative efficacy of SCLC chemotherapy

Actual group	MALDI-TOF-MS classification		Total	Accuracy rate
	Labeled as “Disease response group II”	Labeled as “Disease progression group II”		
Disease response group II	27	3	30	91.4%
Disease progression group II	2	26	28	

### Survival analysis

**Fig. 3:**
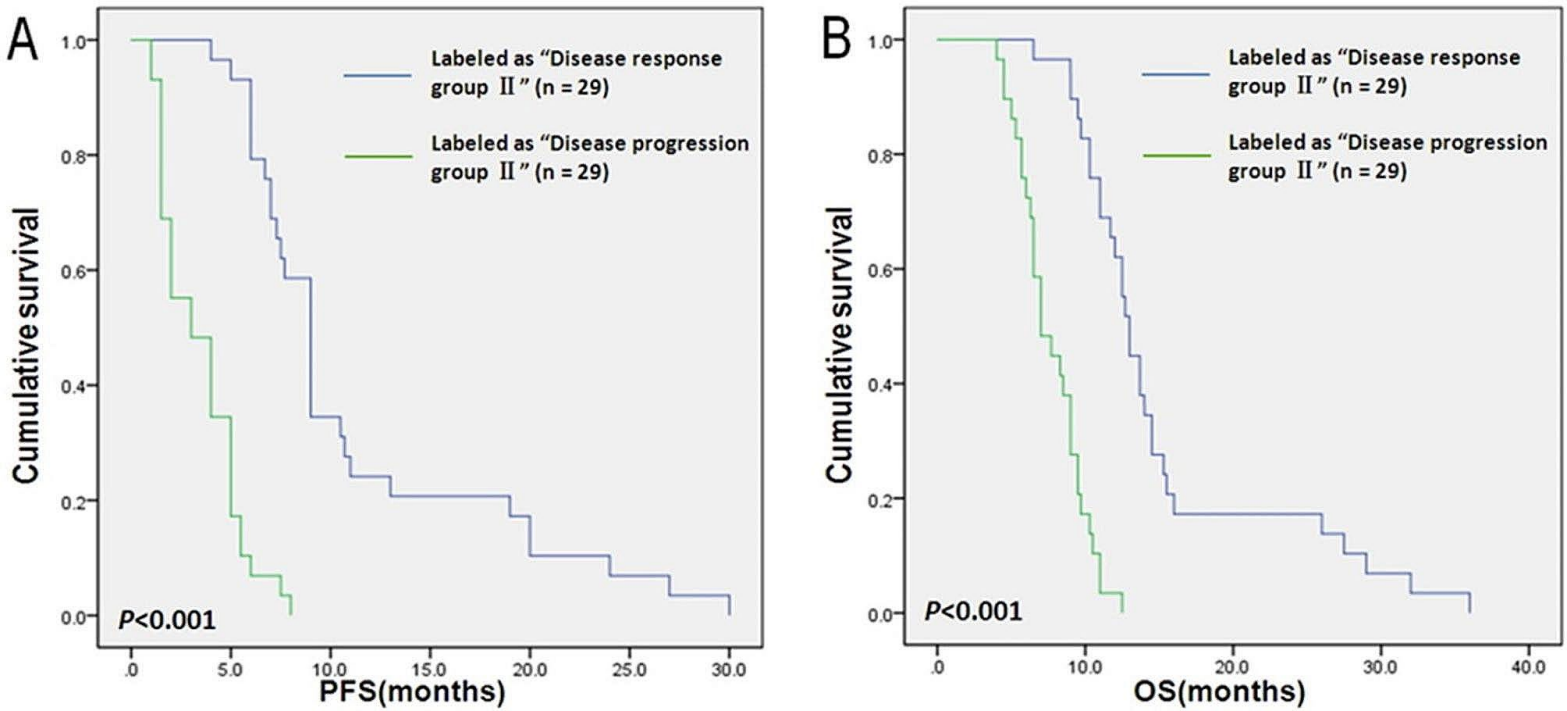
Survival analysis of 58 patients in the validation group. (**A**) 29 patients were labeled as “Disease response group II” had obviously inferior PFS (*P* < 0.001) when compared to 29 patients were labeled as “Disease progression group II”. (**B**) 29 patients were labeled as “Disease response group II” had obviously inferior OS (*P* < 0.001) when compared to 29 patients were labeled as “Disease progression group II”

The Kaplan-Meier survival plots of PFS and OS for patients whose matched samples were labeled as “Disease response group II” or “Disease progression group II” by the classifier are shown in Fig. 3. The median PFS of patients whose matched samples were labeled as “Disease response group II” and “Disease progression group II” by the classifier were 9.0 months (95%CI: 8.1 to 9.9) and 3.0 months (95%CI: 1.2 to 4.8), respectively. Patients whose matched samples were labeled as “Disease response group II” by the classifier had a significantly longer PFS than patients whose matched samples were labeled as “Disease progression group II” (χ^2^ = 46.98, *P* < 0.001, log-rank test, Figgure 3 A). Patients whose matched samples were labeled as “Disease response group II” by the classifier had an OS time of 13.0 months (95%CI: 12.1 to 13.9), compared with 7.0 months (95%CI: 5.4 to 8.6) for the patients whose matched samples were labeled as “Disease progression group II”, showing the statistically significant differences between two groups (χ^2^ = 40.64, *P* < 0.001, log-rank test, Fig. 3B).

## Discussion

SCLC is a highly malignant tumor featuring poor cell differentiation, which is generally sensitive to chemotherapy. As a result, chemotherapy is the cornerstone treatment for SCLC, and the efficacy of first-line chemotherapy is positively related to the PFS and OS of the patient [18]. Therefore, timely administration of first-line chemotherapy regimen with the best efficacy enables the achievement of longer PFS, higher survival and extended OS. The first-line standard chemotherapy regimen recommended by guidelines is the combinative protocol of platinum-based drugs and etoposide (EP regimen). It has been reported that the objective response rate of the EP regimen for the treatment of extensive stage SCLC is approximately 75-85% [19] and the median OS is 8 ∼ 13 months. Although they have a relatively high response rate, a portion of SCLC patients remain in disease progression during first-line chemotherapy, resulting in the failure of the treatment and impacting the patients’ survival. Accordingly, predicting the curative efficacy prior to treatment has been frequently studied in recent oncological clinical research. It is suggested that the occurrence of primary drug resistance, especially multidrug resistance (MDR), is the critical cause of failure of initial SCLC treatment through a variety of known and unknown mechanisms [20]. It is very difficult to apply pertinent treatment strategies that target each mechanism of drug resistance in clinical practice; however, all of the mechanisms will induce alteration of the proteome. Consequently, proteomics, and comparative proteomics in particular, provide new solutions to finding potential tumor markers related to oncologic drug resistance.

Proteomic technologies mainly include protein isolation techniques such as two-dimensional polyacrylamide gel electrophoresis (2-D PAGE) and protein identification techniques such as mass spectrometry. These techniques play a critical role in the development of tumor marker studies, and the latter technique combines high-throughput proteomics research with clinical practice, allowing analysis of many samples with extremely high sensitivity, which can be applied to discover potential markers for early diagnosis of many diseases [21, 22]. In recent years, the mass-spectrometric technique has been widely used in the detection of various proteins and has been continuously developed for histological and serological studies of many tumors, among which lung carcinoma is the hot topic in both clinical and theoretical research [23–25]. Yanakisawa [26] reported that the proteomic analysis of NSCLC tissues could predict the pathological types, disease stage and prognosis. Taguchi [8] and Yang [27] et al. reported that serum proteomic types of NSCLC patients could be used to predict the curative efficacy of gefitinib or erlotinib as well as the survival conditions. This technique is mature and has been commercialized. Han et al. [28] used surface-enhanced laser desorption ionization-time of flight-mass spectrometry (SELDI-TOF-MS), with an SVM calculation method (Support Vector Machine) to analyze the recent efficacy of a first-line combinative chemical regimen of cisplatin and VP-16 for SCLC. This study established a model with 92.4% sensitivity and 92.5% specificity and an efficacy prediction model using two mass spectrum peaks with mass-to-charge ratios of 8830 Da and 10,568 Da by comparing the serum mass spectrum changes before and after treatment. This model differentiated the drug resistance and chemotherapy-sensitive groups with an 80.0% accuracy rate. In light of the progress in the use of mass-spectrometric techniques in oncological research, we adopted the MALDI-TOF-MS technique to analyze the serum protein/peptides of patients in two groups with different curative efficacies in first-line chemotherapy and used the varying protein/peptides to establish the classification model. We aimed to achieve the objective of predicting the curative efficacy of first-line chemotherapy in SCLC, which was novel in SCLC studies related to chemotherapy efficacy prediction. This study adopted the ClinPro™ detecting system (Brooke Dalton Company, Germany), which consolidated the systems, including magnetic bead separator protein/peptides, MALDI-TOF-MS, and built-in analysis software. This system had both the characteristics of salt tolerance and pollution resistance and showed high sensitivity, repeatability and reproducibility. As the instrument was of high resolution, its accuracy could achieve a 10 ppm level in a stable and reliable manner when small molecular protein/peptides of approximately 2,000 Da were tested. In addition, liquid magnetic beads could fully combine low-abundance proteins or protein/peptides, which allowed for smaller samples and a faster analysis process to realize standardization and high throughput [29]. The major process of the experiment was as follows: prepare nano chelating magnetic beads to remove the high-abundance proteins and impurities from the samples and aggregate low-abundance proteins and bind them to the magnetic beads, elute target proteins or protein/peptides from the magnetic beads and mix them with matrix, dot on the target board that was placed in the instrument for flight-mass spectrometry detection, obtain the fingerprints of each sample, use the built-in software to analyze the graphs to discover protein/peptides that differ between groups with statistical significance, and seek the optimal combination of protein/peptides with a biological calculation method to establish the model, which was finally blinded validated for its sensitivity, specificity and accuracy. This technique has been widely applied in large clinical centers in foreign countries for early diagnosis and clinical research of various tumors [30–34].

Through this system, we analyzed the pre-treatment serum samples of two groups of patients who had differing efficacies of first-line standard chemotherapy to discover the protein/peptides that varied between groups and establish the model. The efficacy prediction model, established using a biological calculation method (GA method), included four serum protein/peptides that had significantly different expression levels between the two groups. By blinded validation, the overall accuracy of our model was 91.4% (53/58). To verify whether this model could predict prognosis, we conducted a survival analysis for patients in the validation group. These patients were classified into Disease response and Disease progression groups. The results revealed that both the median PFS and median OS of SCLC patients in the Disease response group were significantly longer than those in the disease progression group, showing a remarkable benefit in survival for patients in the former group. Due to the limited sample size for survival analysis, we could not perform a multivariate survival analysis for these patients. However, the survival curves showed significant differences between the two groups, indicating that this model could be used for preliminary prognosis prediction for SCLC patients undergoing first-line chemotherapy.

Compared with other types of samples such as tissues, sputum and pleural effusion, blood samples can be easily collected and managed in clinical practice for use as study materials. We can obtain the samples at any time during different treatment stages. As the overall surface area of the nano chelating magnetic beads is large, it can specifically combine the low-abundance proteins in the samples and capture more types of protein/peptides without loss of the protein/peptides during removal of the high-abundance proteins by other methods, keeping the high sensitivity and specificity of the system [35, 36]. When tumor tissues exist in human body, the composition of serum proteins and protein/peptides will change due to even minor changes in genes, metabolism and internal environment. In our study, we took advantage of high sensitivity and specificity advanced comparative proteomics technology to compare the serum samples of different patients and then constructed an efficacy prediction model for SCLC patients based on the protein/peptides mass spectrum peaks observed. This model could forecast the curative efficacy rapidly and accurately for SCLC patients undergoing first-line standard chemotherapy. Because of the tumor heterogeneity (pathological grading, disease stage, patient’s age, gender, and genetic background), most researchers consider these composite indexes based on biomarkers more valuable than specific single tumor markers in clinical practice [37, 38].

This novel study adopted the MALDI-TOF-MS technique to develop a curative efficacy prediction model for SCLC patients receiving chemotherapy based on protein/peptides composition. Because it was a single-center independent study with limited sample size, studies with a larger sample size at multiple centers are required to fully validate the accuracy of the model. Moreover, the incorporation of other proteomic technologies with bioinformatics technology is necessary to elaborate on the current information about this group of protein/peptides.

## Conclusion

The results of this study showed that the serum protein/peptides were different in SCLC patients with different curative efficacies of chemotherapy. These protein/peptides could be used for the preliminary prediction of efficacy and prognosis of SCLC patients receiving first-line chemotherapy, indicating the possibility of applying this serum protein/peptides classification model in the clinical setting.

## Data Availability

No datasets were generated or analysed during the current study.

## Abbreviations

CR: Complete response
CT: Computed tomography
ECOG: Eastern Cooperative Oncology Group
EGFR-TKIs: Epidermal growth factor receptor tyrosine kinase inhibitors
GA: Genetic algorithm
KM: Kaplan-Meier
MALDI: TOF-MS matrix-assisted laser desorption/ionization time-of-flight mass spectrometry
MDR: Multidrug resistance
NSCLC: Non-small-cell lung cancer
OS: The median overall survival
OS: Overall survival
PD: Progressive disease
PFS median progression: Free survival
PR: Partial response
QC: Quick classifier algorithm
RR: The response rate
SCLC small: Cell lung cancer
SELDI-TOF-MS: Surface-enhanced laser desorption ionization-time of flight-mass spectrometry
SNN: Supervised Neural Network
VP-16: Etoposide
